# Standardized ultrasound evaluation of carotid stenosis for clinical trials: University of Washington Ultrasound Reading Center

**DOI:** 10.1186/1476-7120-8-39

**Published:** 2010-09-07

**Authors:** Kirk W Beach, Robert O Bergelin, Daniel F Leotta, Jean F Primozich, P Max Sevareid, Edward T Stutzman, R Eugene Zierler

**Affiliations:** 1 D. Eugene Strandness Vascular Laboratory, Department of Surgery, University of Washington, Seattle, Washington 98195, USA

## Abstract

**Introduction:**

Serial monitoring of patients participating in clinical trials of carotid artery therapy requires noninvasive precision methods that are inexpensive, safe and widely available. Noninvasive ultrasonic duplex Doppler velocimetry provides a precision method that can be used for recruitment qualification, pre-treatment classification and post treatment surveillance for remodeling and restenosis. The University of Washington Ultrasound Reading Center (UWURC) provides a uniform examination protocol and interpretation of duplex Doppler velocity measurements.

**Methods:**

Doppler waveforms from 6 locations along the common carotid and internal carotid artery path to the brain plus the external carotid and vertebral arteries on each side using a Doppler examination angle of 60 degrees are evaluated. The UWURC verifies all measurements against the images and waveforms for the database, which includes pre-procedure, post-procedure and annual follow-up examinations. Doppler angle alignment errors greater than 3 degrees and Doppler velocity measurement errors greater than 0.05 m/s are corrected.

**Results:**

Angle adjusted Doppler velocity measurements produce higher values when higher Doppler examination angles are used. The definition of peak systolic velocity varies between examiners when spectral broadening due to turbulence is present. Examples of measurements are shown.

**Discussion:**

Although ultrasonic duplex Doppler methods are widely used in carotid artery diagnosis, there is disagreement about how the examinations should be performed and how the results should be validated. In clinical trails, a centralized reading center can unify the methods. Because the goals of research examinations are different from those of clinical examinations, screening and diagnostic clinical examinations may require fewer velocity measurements.

## Background

Repair of carotid artery stenoses (carotid revascularization) has been shown to be effective in reducing the chance of embolic stroke from carotid plaque rupture and embolization to the brain [1]. Clinical trials of carotid artery revascularization methods such as carotid endarterectomy and carotid artery stenting are in progress to provide guidance to clinicians about the choice of therapy.

Noninvasive ultrasonic duplex Doppler examination has been a standard method for the clinical evaluation of the carotid arteries for a third of a century [2,3]. Doppler velocity waveforms are gathered from the common and internal carotid arteries to detect local elevated blood velocity as a marker of arterial stenosis allowing categorical classification of the right and left common and internal carotid arteries into clinically useful categories. One often used classification scheme is: 1) no significant stenosis (< 50%DR), 2) moderate stenosis (50%-79%DR), 3) severe stenosis (80%-99%DR), and 4) occluded. The method and associated criteria for stenosis classification were developed in the decade prior to 1990 [3-10]. The reference standard for the classification method is X-ray contrast angiography. Some publications use other angiographic categories with divisions at 60%, 70% or other values.

A variety of Doppler velocity measurement methods are used to classify arteries into the proper angiographic categories. However, detailed publications demonstrate that although satisfactory sensitivities and specificities can be obtained by associating selected angiographic classifications with particular Doppler measurements, the relationship between Doppler measurements and angiography is not a narrow monotonic line, [11] but a multivariate relationship. The additional variables include: the presence of a moderate or severe contralateral stenosis [12-15], cerebral territory perfused [16], completeness of the circle of Willis [17-19], ipsilateral collateral flow [20], vertebral flow [21] and method of revascularization [22].

## Methods

All carotid ultrasound duplex Doppler examinations are performed by field centers under IRB approval at the field center institutions. A duplex Doppler Ultrasound Protocol Manual is provided to each participating ultrasound laboratory by the University of Washington Ultrasound Reading Center (UWURC). Anonymized images and worksheets from each examination are sent to the UWURC.

**Figure 1:**
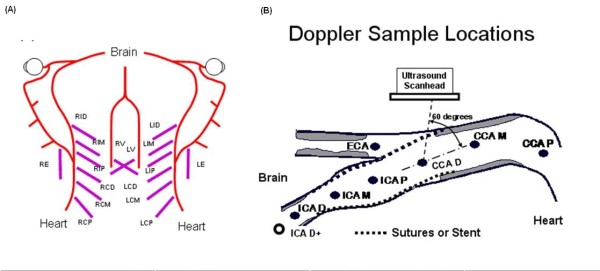
**Doppler Waveform Locations**. A. Map of the arterial system from the chest (bottom) to the brain (top) showing the proximal (P), middle (M) and distal (D) locations in the right (R) and left (L) common (C), and internal (I) carotid plus the external carotid (E) and vertebral (V) arteries, using short abbreviations. B. Recommended Doppler locations in the common and internal carotid arteries were separated by between 1 and 2 cm with the ICA/ECA flow divider as the key landmark, showing common abbreviations.

The protocol specifies that at least 16 ultrasound B-mode images with associated Doppler waveforms be gathered from each patient: On each side the sonographer should acquire 3 images from the common carotid artery (CCA), 3 from the internal carotid artery (ICA), one from the external carotid artery (ECA) and one from the vertebral artery (VA) (Figure 1). Additional images and waveforms are required from locations distal to the stent (to detect post-stent stenosis) and distal to any stenosis (to document post-stenotic turbulence).

For each of the 16 or more spectral waveforms, systolic and diastolic velocities are measured and transcribed along with the Doppler angles (from the associated B-mode images) to a standard worksheet. The worksheet is submitted with paper, film, photocopied or electronic versions of the images to the UWURC. Studies on video tape recordings are discouraged because of the excessive time required for video processing.

**Figure 2:**
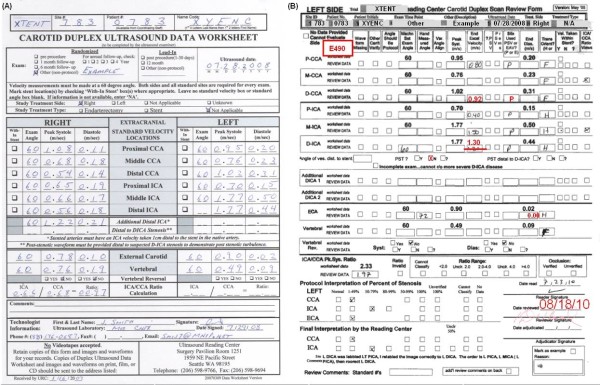
**Duplex Data Worksheet and Review Form**. A. The Duplex Data Worksheet is submitted to the UWURC by the sonographer plus copies of the images to document the duplex Doppler data. B. UWURC Review Form with keyed data and reader/reviewer entries. Black printed entries are keyed from the worksheet submitted from the field center. Hand written entries by the reader were transcribed or measured from the image provided by the field centers. Typed entries in red by the reviewer mark additional changes based on the images.

**Figure 3:**
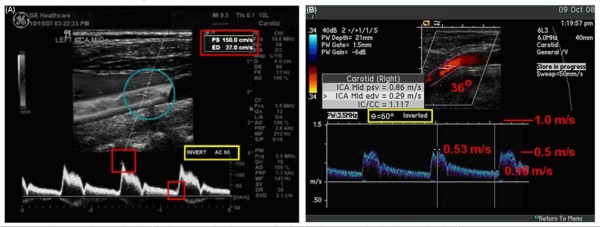
**Normal CCA and ICA Images**. A. This image was supplied to the UWURC on multiformat transparent film and scanned into the electronic image. Numbers in the red and yellow boxes have been enhanced for easier reading. B. The Doppler cursor is not aligned with the artery axis; the Doppler angle is 36°. A new velocity scale has been added in red along the right edge of the spectral waveform showing the results of the Doppler equation for a 36°angle.

**Figure 4:**
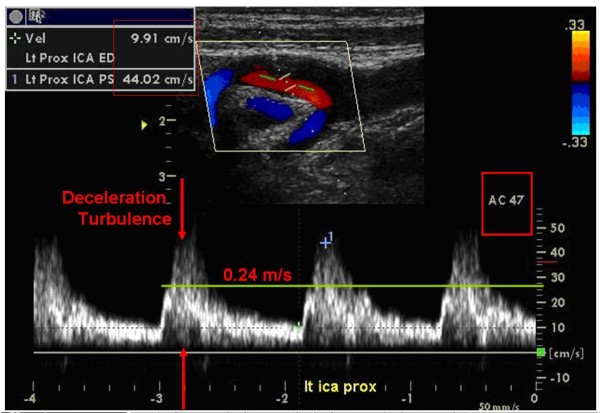
**End Acceleration Velocity Followed by Deceleration Turbulence**. Cursor 1 was placed by the examiner at the traditional location for peak systolic velocity. The Green line was added by the UWURC Reader at the End Acceleration Velocity; the red annotation was added by the UWURC Reviewer. The Doppler Angle is 47 degrees

At the UWURC, worksheet data (Figure 2A) are single keyed into the UWURC database. For each side of each case, a review form is printed (Figure 2B) including the keyed worksheet data. During UWURC review, the reader verifies the anatomic location of each waveform from the B-mode image labels and anatomic features, determines whether a stent can be seen and verifies correct transcription of the data from the images (Figure 3) including proper location of the decimal points (some images are marked in cm/s, others in m/s). The reader also checks the Doppler angle alignment on the image and the spectral velocity measurement cursors on the waveform and checks for end acceleration velocity (EAV) (Figure 4). If the measurement cursors are absent or improperly placed, the reader marks and measures the velocities and indicates whether the Doppler ultrasound beam was tilted toward the head (H) or foot (F). The reader also marks the preferred waveforms from the common (CCA) and internal (ICA) carotid arteries for use in computing the ratio and classification of stenosis. Finally, the reader checks the computation of the ICA/CCA systolic velocity ratio and marks the classification categories for CCA, ICA and ECA (external carotid artery). After completion of the case by the reader, each value is verified by a reviewer. The completed review form is then sent for double key entry into the UWURC data base.

Turbulence or complicated oscillating flow is most likely to occur during temporal deceleration in the late phase of systole and during spatial deceleration just distal to a stenosis. This turbulence causes bruits or murmurs that can be heard with a stethoscope, and appears as spectral broadening that can be visualized in the spectral waveform. Application of "angle correction" to the Doppler frequency measurement based on the Doppler equation by measuring the Doppler angle between the ultrasound beam and the artery axis is not appropriate for turbulent wavforms because the heading of the velocity vector is random or at least chaotic during spectral broadening. Thus, some examiners differentiate Peak Systolic Velocity (PSV), which is measured during spectral broadening, from End Acceleration Velocity (EAV), which is measured just before the onset of turbulence (Figure 4). Because the PSV is often greater than the EAV and there is no guidance in the literature on which to choose, the UWURC enters both values on the review form for later analysis to provide a basis for selecting one or the other.

**Figure 5:**
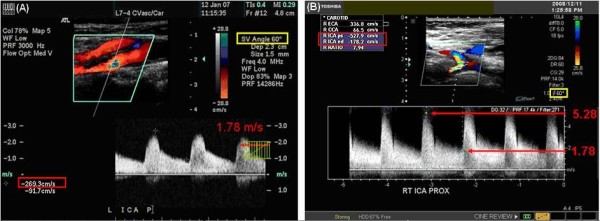
**Stenotic ICA Images and Waveforms**. A. Systolic velocity > 1.25 m/s is consistent with an angiographic stenosis > 50% diameter reduction (DR). Both the PSV = 2.69 m/s and the EAV = 1.78 m/s exceed the criterion. Color aliasing (cyan/blue) can be seen at the stenosis location, although the color aliasing velocity of 0.29 m/s, when adjusted for Doppler angle of 60 degrees (0.58 m/s) and for estimated aliasing magnitude (1.16 m/s), suggests that the color image was captured in diastole, at the right edge of the waveform. B. EDV > 1.4 m/s is consistent with a severe angiographic stenosis > 80% DR. A magnified view of the color flow lumen image appears to reveal no hemodynamic stenosis. The apparent filling of an extra-luminal space with color is sometimes due to specular (mirror-like) reflection of ultrasound from the deep luminal surface. If the extravascular tissue is echogenic, write priority will suppress the Doppler color, but if the extravascular tissue is anechoic, Doppler color remains in that portion of the image. The dark (anechoic) region between the color flow lumen and the brighter arterial wall deep to the Doppler color would be interpreted by some as plaque burden.

Two classification methods are used by the UWURC to complete the review form: 1) the ICA/CCA ratio [23] and 2) the "Strandness Criteria" [24,25]. For the ICA/CCA ratio, the EAV is used for each value if available; otherwise, the PSV is used. If both velocities were measured with valid Doppler examination angles between 58 and 61 degrees, then the ratio is calculated; otherwise, an estimate of the ratio is placed into one of 5 categories (less than 2.0, near 2.0, between 2.0 and 4.0, near 4.0, greater than 4.0), or cannot classify. The ratio criterion 2.0 separates stenoses < 50% from those > 50% [26]; the ratio criterion 4.0 defines the 70% stenosis boundary [23]. The "Strandness" velocity criteria separate stenoses at: 1) the 50% (ACAS) boundary with a PSV criterion of 1.25 m/s [24,25] and at 2) the 80% (ACAS) stenosis boundary with an EDV criterion of 1.4 m/s [27] (Figure 5). Because of the plethora of classification methods for both angiography and for duplex Doppler, with indistinguishable sensitivity and specificity measures, the UWURC refers to stenoses simply as moderate or severe.

**Figure 6:**
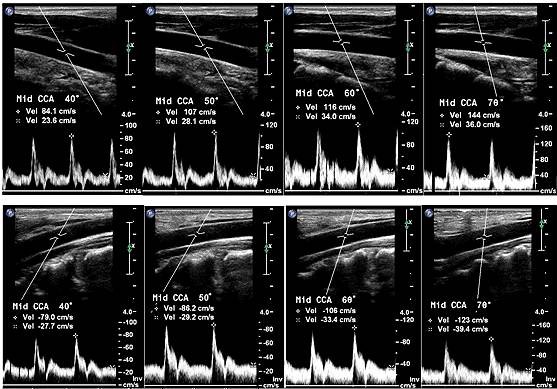
**Effect of Doppler Examination Angle**. All 8 images were acquired from the same location in the same artery within the same examination period using the Doppler equation to correct for the geometric angle between the ultrasound beam and the artery axis. All duplex Doppler ultrasound instruments use this method to adjust the velocity measurement value reported. This systematic measurement bias (higher values for larger angles) appears in all peripheral arteries. The sole exception is measurements distal to long straight arteries such as the distal superficial femoral artery. For equal Doppler angles, steering the ultrasound beam toward the feet (upper row) appears to provide similar values to steering the ultrasound beam toward the head (lower row), if the same Doppler angle is used, but the effect of this variable should be quantified.

**Figure 7:**
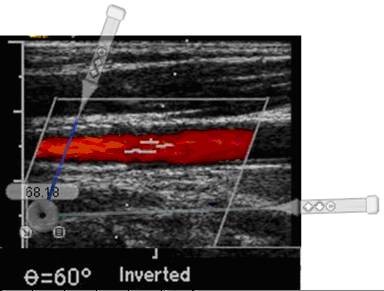
**Doppler angle cursor misaligned with artery axis**. Angle remeasured with "Screen Protractor" [30]. The protractor is moved over the image to align one protractor cursor with the Doppler beam cursor and the other protractor cursor with the artery axis. Here the protractor has been moved aside to allow easy visualization of the original image. The proximal end of the stent is visible in the left half of this image.

The vascular diagnostic community is divided into two groups: 1) those that perform duplex Doppler examinations using a 60 degree Doppler angle between the ultrasound beam and the vessel axis, and 2) those that use a convenient angle less than or equal to 60 degrees [28]. Both groups then apply a geometric adjustment using the Doppler equation (assuming velocity parallel to the artery axis) to compute the arterial velocity. However, normal arterial flow is not usually parallel to the artery axis [29], thus the assumption behind the Doppler equation is not valid. Attempts to validate the Doppler equation in normal carotid arteries (Figure 6) and stenotic arteries result in a systematic bias: 1) using larger Doppler examination angles result in higher velocity values and 2) the relationship is monotonic. To minimize the effect of different Doppler angles between visits, the UWURC has recommended that, whenever possible, carotid artery Doppler ultrasound measurements are acquired at a Doppler examination angle of 60 degrees. The UWURC has accepted and evaluated all submitted ultrasound examination velocities, including those taken at Doppler examination angles other than 60 degrees. For measurements with incorrect angle measurement alignment visible on the B-mode image (Figure 7), the UWURC remeasured the angle using a protractor overlying the image [30] and entered the remeasured angle in the database so that the appropriate geometric correction could be applied (Figure 3B) before analysis.

The resulting data form (Figure 2B) accommodates five final numeric values for each of the 16 recommended and 2 optional (additional distal ICA) measurements: 1) "Machine Set Angle" (MSA), 2) "Hand Measured Angle" (HMA), 3) "Peak Systolic Velocity" (PSV), 4) "End Acceleration Velocity" (EAV), 5) "End Diastolic Velocity" (EDV). There are also ten categorical values: 1) Waveform Missing, 2) Other Can't Verify (when the anatomic location cannot be established), 3) Angle should be Protocol (when a Doppler angle other than 60 degrees is used but a 60 degree angle could have been used), 4) Variable Angle Alignment (when the Doppler sample volume is located in a curve or other anatomic location in which the angle could have been measured differently) 5) ?PSV (when due to arrhythmia or to turbulence (spectral broadening) the systolic velocity value is uncertain, 6) PSV remeasured (used as an interim variable for marking EAV on a prior version of the review form), 7) PSV or EAV (marks whether the examiner measured the PSV or EAV), 8) H or F (marks whether the Doppler cursor was angled toward the head or the foot), 9) Velocity in Stent (provides an indication of stent location), 10) Ratio View (marks the CCA and ICA values used in computing the velocity ratio).

Each of these variables is designed either to document a feature of the measurement or to provide the basis of testing specific hypotheses in future publications. For values marked "Waveform Missing" or "Can't Verify", the values may contain errors not detected by the review process because of missing or obscured images. For instance, if a study was submitted as a clinical report, with some velocity values reported as text, but no images or waveforms were provided, the clinical values were entered on the Review Form but the corresponding review form lines were marked "Waveform Missing".

After the "Reader" checked all of the data on each form against the source data, the "Reviewer" rechecked all of the data and each reader entry against the source data. Disagreements between Reader and Reviewer were adjudicated by committee to assure uniform reading and reviewing.

**Figure 8:**
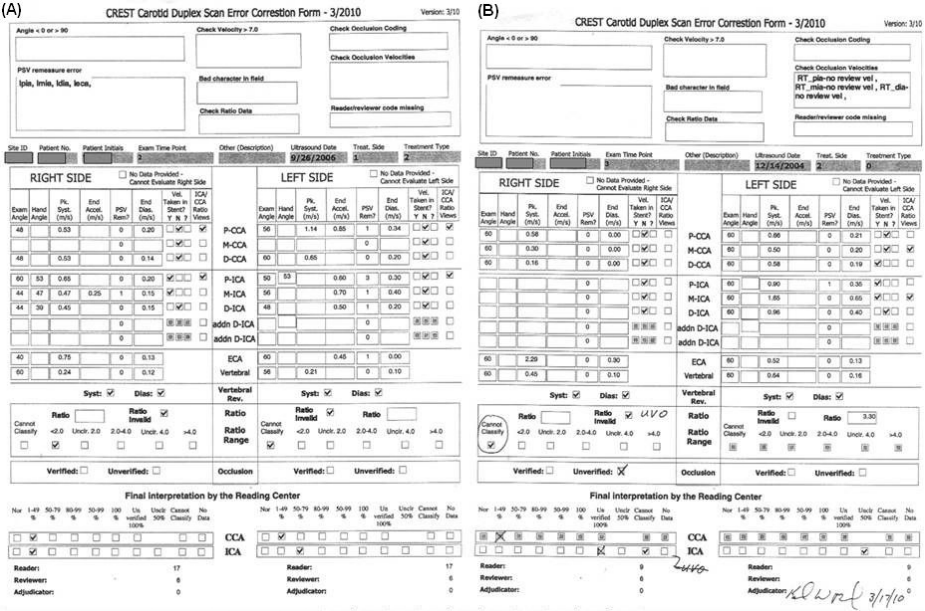
**Error Checking Forms**. A. No PSV values in the left PICA, MICA, DICA & ECA. Most likely cause is that the examiner entered the EAV for these values on an early version of the review form which did not include entry spaces for PSV and EAV separately. B. No velocity values are entered for the occluded Right ICA. In some Case Report Forms, "Occluded" was written into a space intended for the numeric value of 0.00. By policy, for locations marked on the Duplex Data Worksheet with Occl, the reviewer should mark 0.00 for PSV and EDV as a synonym for Occl. If the report states that the vessel is occluded but no entry or images are provided for the measurement location, the velocity entries were left blank, and "unverified occlusion" was marked as the interpretation

Data were compiled into a file and checked for implausible values including: EDV > PSV, EAV > PSV, Angle > 90 degrees, PSV > 6 m/s, and missing values. Such values might pass undetected through the system due to decimal point errors, conversion from alpha to numeric values, and clerical errors. For each case with implausible values, a custom error form was printed (Figure 8) so that the source data could be retrieved, the case re-read and all errors fixed.

All entries keyed into the database are logged to document the Reader, Reviewer, Adjudicator, and Keyer.

## Results

**Figure 9:**
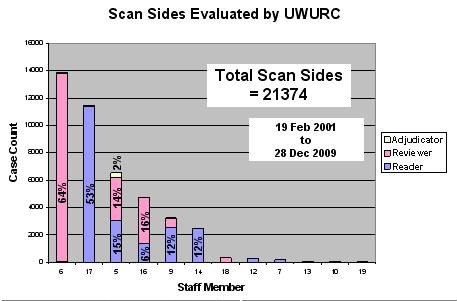
**Evaluation Tabulation**. Number and percentage of review forms evaluated by each reader and reviewer.

Between 1999 and 2009, the UWURC evaluated 10,687 duplex Doppler examinations comprised of 21,374 sides (Figure 9). 12 staff members were qualified to evaluate examination images. 53% of the examinations were read by a single staff member; an additional 45% were read by 4 others. 64% of the examinations were reviewed by a single reviewer; an additional 30% were reviewed by two others. Fewer than 3% of the examination sides required adjudication. In no case was the reader and the reviewer the same person; the signing adjudicator could be either reader or reviewer.

**Figure 10:**
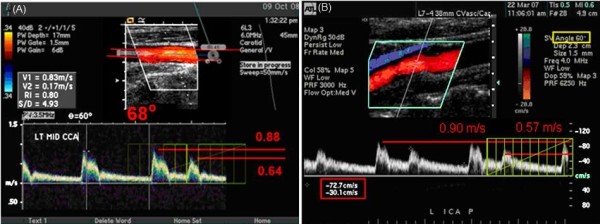
**Arrhythmia Doppler Waveforms**. Note the variability in systolic velocities with different preceding diastolic periods.

Although the majority of waveforms and images were easily interpreted and classified, in some images the selection of a correct measurement required discussion. In the case of an arrhythmia (Figure 10), the systolic velocities following a long diastolic period have elevated values compared to those following a short diastolic period, because increased ventricular filling during the longer diastole elevates the ventricular ejection volume. This variation in systolic velocity causes uncertainty in the measurement, and affects derived systolic ratio measurements. In such cases a ?PSV entry is made on the review form. When possible, measurements within a study are taken at each location from a systole following a "normal" diastolic interval.

**Figure 11:**
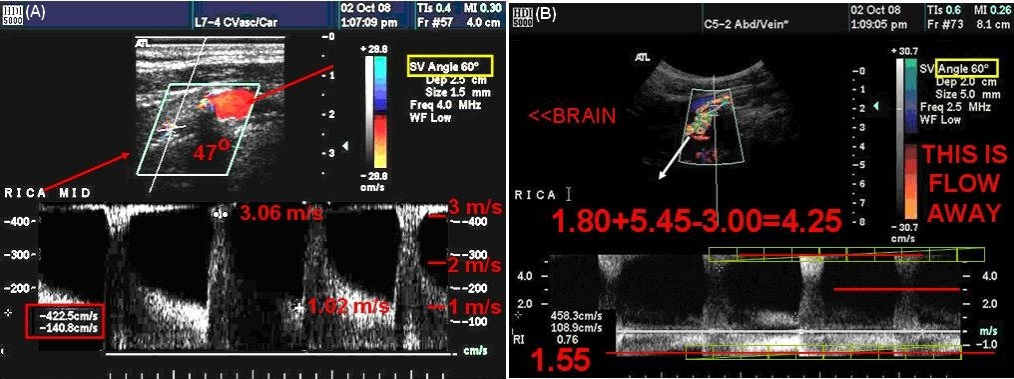
**Severe Stenosis Misclassification**. A. Misalignment of the Doppler cursor, yielding an elevated angle measurement, results in an EDV > 1.4 m/s, indicating a severe stenosis within this stent. Proper cursor alignment would result in EDV = 1.02 m/s, indicating a moderate stenosis. Note the aliased systolic peak extending to the 100 cm/s value. After correcting for aliasing and for angle misalignment, the correct systolic velocity is 3.8 m/s. B. Faced with a bidirectional waveform, the examining sonographer measured the reversed (+) portion of the waveform (indicating arterial flow from brain to heart), measuring the EDV as 1.09 m/s and indicating moderate stenosis, rather than the correct 1.55 m/s value indicating severe stenosis. They also measured a PSV of 4.58 m/s rather than the correct 4.25 m/s value.

The correct classification of significant stenoses into moderate or severe categories is most important for both clinical management and for clinical trials surveillance. Sonographic errors, if undetected on evaluation, can result in misclassification. Figure 11 provides two examples of cases misclassified by the sonographer according to the protocol.

**Figure 12:**
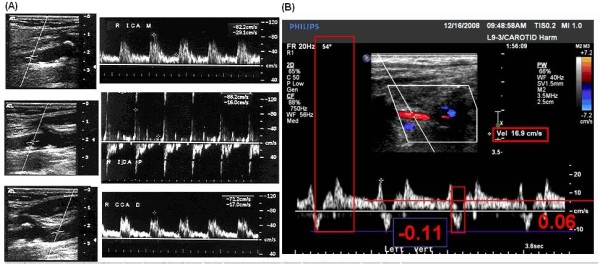
**Velocity Reversal and Flow Reversal**. A. Lower and Upper Normal waveforms indicate a vessel without stenosis. Middle: Complicated velocities in the region of the bifurcation appear dominated by reversed velocity often called "flow separation". B. Systolic reversal in vertebral arteries is a frequent finding. The examining sonographer marked the EAV as the systolic velocity and did not mark an EDV. The UWURC reader marked the negative systolic peak.

Flow reversal in the extracranial arterial system is unusual except in cases of severe stenosis, occlusion, steal or aortic regurgitation. Figure 12 shows examples of velocity reversal. The Doppler waveform "reversal" in figure 12A near the carotid bifurcation could not be flow reversal because the arteries proximal and distal have normal forward waveforms. This is an example of the effect of complicated flow, with velocity toward the transducer (at an angle of 60 degrees to the vessel axis) in one sampled portion of the carotid bulb during temporal deceleration at the end of systole. This is often called "flow separation". This waveform should not be interpreted as indicating net flow in the carotid artery directed from the head toward the heart. The measurement of net flow requires complete sampling of velocities perpendicular to a surface that transects the vessel, and then integrating [velocities*area] to compute instantaneous flow. Unilateral left vertebral systolic flow reversal (Figure 12B) may indicate a stenosis at the origin of the left subclavian artery resulting in subclavian steal [31].

The data have been compiled into a database that can be configured for analysis by patient, side, treatment side, and/or time point to allow longitudinal or cross sectional comparisons.

**Figure 13:**
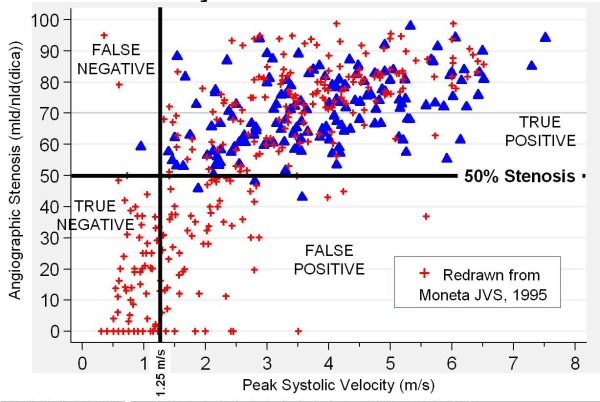
**Doppler Velocity vs**. X-ray Angiography Correlation. Blue Triangles: Values from UWURC substudy [32]. Red Crosses: Values from the literature [11].

The accuracy of duplex Doppler ultrasound is one of the most frequently discussed topics in carotid artery diagnosis. Carotid Doppler velocities are used to classify arteries into stenotic categories. From a subgroup of pre-procedure studies, Doppler velocity values were plotted against angiographic measurements in a small subpopulation [32] (Figure 13) and compared to literature values [11]. Within the range of values available in this clinical trial (blue triangles, Stenosis 42% DR to 98% DR), the relationship does not suggest that systolic velocity would provide good sensitivity or specificity for the clinical classification threshold of 70% DR.

**Figure 14:**
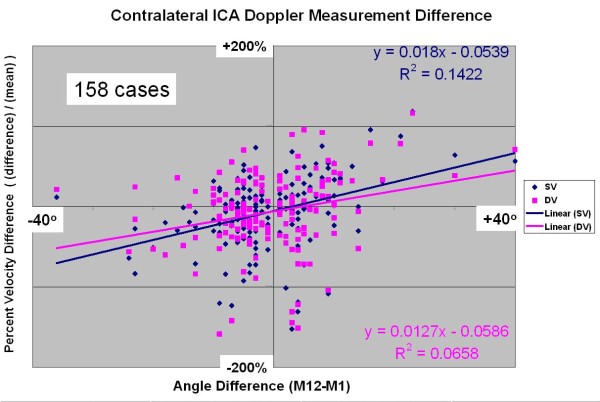
**Percent Velocity Value Change with Doppler Angle Change**.

The choice of Doppler angle is another frequently discussed question [33]: should the Doppler angle be 60 degrees or the smallest angle possible, so long as it is less than 60 degrees? For a subgroup of patients with 1 month and 12 month post-procedure studies, the change in contralateral systolic and diastolic velocities was plotted versus the change in Doppler angle (Figure 14) in cases which used different Doppler angles during the two studies. The positive slope comparing percent velocity change to angle difference is consistent with the experiment shown in figure 6.

## Discussion

The most objective and comprehensive survey of carotid artery examination methods is the 2002 Carotid Ultrasound Consensus Conference [28]. In 1997, the University of Washington Ultrasound Reading Center designed the ultrasound protocol which complies with the recommendations later adopted by the consensus conference, with three exceptions: 1) The UWURC recommends the consistent use of a Doppler examination angle of 60 degrees; the Consensus Conference reports disagreement, with some members recommending 60 degrees and some recommending < 60 degrees; 2) the UWURC Doppler diastolic velocity criterion for severe stenosis is 1.4 m/s rather than 1.0 m/s as recommended by the Consensus Conference; and 3) the UWURC makes no recommendation about the evaluation of B-mode or color Doppler images, except for the identification of the location of a stent at the Doppler sample location; the Consensus Conference recommends the evaluation of these images, but provides no quantitative method of reporting the evaluation.

The Consensus Conference explains "the ability of Doppler ultrasound to ... estimate the degree of stenosis [has] been disappointing." so "Doppler ultrasound cannot be used to predict a single percentage of stenosis." but "..criteria should be consistently applied." "Published literature is replete with velocity thresholds.." "The panel suggested that ICA PSV and the presence of plaque on ... images ... should be used when diagnosing and grading ICA stenosis." "The ICA PSV is easy to obtain, has good reproducibility, and should be used in conjunction with grayscale and color Doppler.." "Two additional parameters, ICA-to-CCA PSV ratio and ICA EDV are useful .... " A summary of recommended criteria are included in Table 3." of the consensus paper [28].

The UWURC agrees with all of the findings, but practices the following minor differences for the classification of severity of stenosis.

1. The UW classifications, established prior to 1990, were based on a lower boundary for severe stenosis of 80% DR by angiography: (NLD-MLD)/NLD where MLD is minimum lumen diameter and NLD is the normal lumen diameter of the carotid bulb (ACAS method). Subsequently, others have adopted a 70% lower boundary where NLD is the normal lumen diameter of the ICA distal to the stenosis (NASCET method). Generally the bulb diameter is 1.5 times the normal distal ICA diameter, thus 70% NASCET stenosis = 80% ACAS stenosis.

2. The consensus paper offers two criteria for the 70% stenosis: PSV = 2.3 m/s and EDV = 1.0 m/s. The UWURC recommends EDV = 1.4 m/s. Because the UWURC includes the velocity values in the database, future analyses can elect to use any of these criteria.

There is also a philosophical difference between the consensus document and the UWURC recommendations. While the consensus document recommends that diagnosis be based on a combination of observations from the grayscale B-mode image, color Doppler and spectral Doppler, the exact method of combination is unclear and the use of multiple variables or observations can lead to conflicting results. The two alternate methods used in the summary portion of the UWURC review form--one based on highest PSV(ICA) with EDV(ICA) and the other based on PSV(ICA)/PSV(CCA) ratio -- will not necessarily agree, and should not be used together, but rather, one method should be selected and used consistently.

The relationship between Doppler velocity and angiographic stenosis within the significant stenosis range of interventional trials is poor. The sensitivity and specificity of the test only improves when a large number of cases with minimal or no stenosis are included in the tabulation. Perhaps we have been naïve in the quest for a linear relationship between Doppler velocity and stenotic diameter. Although each hemisphere of the brain does demand a constant average blood supply, independent of intelligence or occupation, a stenosis is likely to induce flow diversion to other potential collateral pathways (contralateral arteries or the ipsilateral external carotid or vertebral arteries), reducing the trans-stenotic flow and velocity by an unpredictable amount. The pattern of the flow diversion might provide important information for the velocity stenosis relationship, and in addition might allow inferences about recruited collaterals which might serve to reduce the risk of stroke below the chance predicted by the stenosis alone. Some sonographers do report the ratio ((ipsilateral CCA PSV)/(contralateral CCA PSV)) to support the diagnosis of ICA stenosis. However, a value less than 1.0 which indicates stenosis also indicates intracranial collateralization, which might be protective against stroke. Thus, although carotid Doppler has been used clinically for a third of a century, puzzles remain and opportunities to improve the method invite exploration.

The geometry of the Doppler equation predicts that the Doppler frequency shift will be zero if the Doppler angle is perpendicular (90 degrees). However, because of transit-time spectral broadening, helical (laminar) flow and complicated turbulent or eddy flow, even at 90 degrees the envelope of the Doppler frequency shift spectrum is not zero. This broadening affects all of the Doppler measurements except those made at a Doppler angle of zero degrees. Unfortunately, a Doppler angle of zero degrees is not possible in ultrasound examination of peripheral arteries and veins. As a result, all "angle corrected" Doppler velocity measurements monotonically increase with Doppler angle from zero to 90 degrees. If the Doppler frequency in the Doppler equation is held constant, and the Doppler angle is changed from 40 degrees to 60 degrees, the computed Doppler velocity increases by 42% or 2.1% per degree. In Figure 6, the velocity value increases by about 1.5% per degree between the 40 degree measurement and the 60 degree measurement in PSV and one EDV, and in the other EDV measurement by 0.8%. Note that in Figure 1,4, the best fit line for systolic velocity measurement increases by 1.8% per degree and the diastolic velocity measurement increased by 1.27% per degree. These values are consistent with the values that can be estimated from the Figure 1 1.30 in Primozich [24] of 2% per degree. It remains to be determined whether the statistically significant dependence on angle is an important factor affecting surveillance precision.

## Conclusions

Although angle adjusted Doppler velocity measurements can be used to classify the severity of carotid stenosis and to monitor the changes in carotid stenosis over time, these velocity values computed from the measurement of a vector component of the velocity vector adjusted by geometric angle projection are not equal to the velocity components parallel to the vessel axis which contributes to the volumetric flow along the artery. The angle adjusted velocity values can only be used for empirical classification based on published standards, and for time to time comparisons of values within each patient. The classifications are only valid when the acquisition protocol is consistent with the standard.

The Ultrasound Reading Center analysis method for duplex Doppler carotid artery data was developed to address several research questions raised in the consensus document and elsewhere:

1. How much of a change in estimated ICA stenosis should be considered significant?

2. What criteria should be used to assess patients after ICA revascularization?

3. Does the degree of contralateral stenosis affect the ipsilateral diagnostic criteria?

A detailed analysis of the data in the future will address these questions and the results will be published.

Because the classification of stenosis into angiographic categories by Doppler has limitations, using this categorical variable for surveillance of a revascularized artery to measure durability can lead to erroneous results. In this case, if a stenosis changes from a "moderate stenosis (50%-79%DR)" to a "severe stenosis (80%-99%DR)", the change in classification might be due to an increase in EDV from 1.38 m/s to 1.42 m/s. Such a small change in measurement might not indicate a change in arterial morphology. An alternative might be to require a change in classification from "no significant stenosis (< 50%DR)" to "severe stenosis (80%-99%DR)", which would be a change from PSV < 1.25 m/s to an EDV > 1.4 m/s. Important progression of a stenosis might not be detected if that were the criteria. If, however, the standard deviation (SD) of the difference in PSV or EDV between visits is measured, then an increase in value more then 3 SD would provide a 99% confidence that the stenosis has become more severe. In the absence of treatment, a decrease in value more than 3 SD would be surprising. However, in a trial of 1000 cases, that rare event would be expected in 10 cases, due t measurement variability rather than stenosis regression.

Research examinations are exploratory, designed to answer a variety of questions. Usually, only a portion of the data gathered in a research protocol is found to be relevant to the questions finally addressed. In contrast, clinical examinations should be designed to efficiently determine whether each patient has a specific treatable condition and whether treatment is likely to improve their quality of life. To refine advice on clinical examination methods, the UWURC will compare pairs of Doppler velocity measurements acquired under the research protocol to address the following questions in future publications:

1) Are three velocity measurements in the CCA necessary to:

a. identify CCA disease?

b. provide a reference denominator for ICA/CCA ratio calculation?

2) Are measurements in the ECA and VA important to the clinical evaluation?

3) Do contralateral velocities decrease when an ipsilateral stenosis is treated suggesting that:

a. intracranial cross-collaterals are present?

b. ipsilateral intra-stenotic velocities might be reduced due to collateral flow?

4) Are particular velocity values or ratios predictive of complications during revascularization?

The first two questions relate to potentially simplifying the clinical examination by omitting superfluous measurements. The third question addresses a cofactor in the correlation between Doppler velocities and angiographic arterial diameter measurements. The fourth question suggests that additional inferences might be derived from a complete clinical examination including modulating the predicted risk of stroke.

Of course clinical carotid examination should be divided into two examinations: 1) screening examinations with a high sensitivity and acceptable specificity for internal carotid artery stenosis which can be carried out in a non-specialist primary care setting, and 2) diagnostic examinations with high specificity for severe carotid stenosis with "vulnerable" plaque to assure that high risk patients are directed to appropriate treatment.

When carotid examinations according to protocol have not been available, the UWURC has accepted data from "clinical examinations" to complete time points in the data set. The minimum data included in the studies have been single velocity measurements from the ICA and CCA on the evaluated side. Demonstration of a single end diastolic carotid velocity exceeding 1.4 m/s is universally accepted as proof of carotid stenotic disease, but verifying a non-stenotic carotid bifurcation requires more documentation.

## Abbreviations

ACAS: Asymptomatic Carotid Atherosclerosis Study; CCA: Common Carotid Artery; DR: Angiographic stenotic Diameter Reduction; EAV: End Acceleration Velocity; ECA: External Carotid Artery; EDV: End Diastolic Velocity; F: Doppler beam directed toward the feet, normal flow "toward" transducer; H: Doppler beam directed toward the head, normal flow "away" from transducer; HMA: Hand Measured Angle by the UWURC from the B-mode image; ICA: Internal Carotid Artery; MSA: Machine Set Angle of the sonographer selected Doppler cursor; NASCET: North American Symptomatic Carotid Endarterectomy Trial; PSV: Peak Systolic Velocity; UWURC: University of Washington Ultrasound Reading Center; VA: Vertebral Artery.

Abbreviations prefixes

D: Distal; L: Left; M: Middle; P: Proximal; R: Right; ? Value uncertain due to arrhythmia.

## Competing interests

The authors declare that they have no competing interests.

## Authors' contributions

KWB Principal Investigator designed methods, reviewed studies, analyzed data and wrote the text. ROB provided data management and analysis. DFL provided data analysis. JFP designed UWURC methods and reviewed studies. PMS provided data analysis. ETS read a majority of the UWURC studies. REZ Clinical Director of the UWURC, provided clinical oversight for the center, analysis and publication. All authors have read and approved the final manuscript.

## Authors' information

Kirk W. Beach, Ph.D., M.D. Emeritus Professor of Surgery and Bioengineering.

Robert O. Bergelin, M.S., Director of Departmental Computing

Daniel F. Leotta, Ph.D., Research Engineer, Applied Physics Laboratory

Jean F. Primozich, B.S., R.V.T., Lead Vascular Technologist

P. Max Sevareid, M.P.H., Project Manager

Edward T. Stutzman, B.S., R.V.T, Vascular Technologist

R Eugene Zierler, M.D., R.V.T., Professor of Vascular Surgery

Department of Surgery

University of Washington,

Seattle, WA 98195

kwbeach@uw.edu
